# A simple and effective protocol for fast isolation of human Tenon’s fibroblasts from a single trabeculectomy biopsy – a comparison of cell behaviour in different culture media

**DOI:** 10.1186/s11658-017-0034-4

**Published:** 2017-03-09

**Authors:** Agata Przekora, Tomasz Zarnowski, Grazyna Ginalska

**Affiliations:** 1 Department of Biochemistry and Biotechnology, Medical University of Lublin, Chodzki 1 Street, 20-093 Lublin, Poland; 2 Department of Ophthalmology, Medical University of Lublin, Chmielna 1 Street, 20-079 Lublin, Poland

**Keywords:** Glaucoma, Ocular disorders, Cell banking, Isolation protocol, Primary culture

## Abstract

**Background:**

Human Tenon’s fibroblasts (HTFs) play a crucial role in wound healing. They cause postoperative scarring of the filtering bleb and are thus responsible for trabeculectomy failure. This study aimed to find an effective and fast protocol for HTF isolation from trabeculectomy biopsies. The protocol was compared with the commonly recommended HTF isolation procedure, which uses Dulbecco’s modified Eagle’s medium (DMEM). We used Eagle’s minimum essential medium (EMEM) enriched with fibroblast growth factor (FGF), which selectively promoted the proliferation of HTF cells. A secondary goal was to compare HTF morphology, metabolism and growth during parallel cultivation of the isolated cells in FGF-enriched EMEM and DMEM.

**Results:**

Standard procedures for HTF isolation from tissue biopsies require a 20- to 30-day culture of the explants to obtain the first monolayer. Our protocol yielded the first monolayer after approx. 15 days. More importantly, the majority of the cells were fibroblasts with only individual epithelium-derived cells present. Using FGF-enriched EMEM allowed 1.3 × 10^6^ vimentin-positive fibroblasts to be obtained from a single biopsy within approx. 25 days. Using DMEM resulted in isolation failure and required exchange to FGF-enriched medium to recover the fibroblast culture. HTFs maintained in FGF-enriched EMEM also showed faster proliferation and a different type I collagen production ability compared to HTFs cultured in DMEM. Thus, FGF-enriched EMEM is recommended for fast propagation of HTFs unless the aim of the study is to assess the effect of a tested agent on proliferation ability or type I collagen production.

**Conclusions:**

Our fast protocol for HTF isolation allows easy setup of cell banks by researchers under laboratory conditions and could be very useful during testing of novel ophthalmologic anti-fibrotic agents in vitro. Molecular analysis of HTFs isolated from patients with known treatment histories may provide valuable information on the effects of some medications taken before glaucoma surgery on the subsequent wound-healing process and potential for trabeculectomy failure.

**Electronic supplementary material:**

The online version of this article (doi:10.1186/s11658-017-0034-4) contains supplementary material, which is available to authorized users.

## Background

Glaucoma is a group of human ocular disorders characterized by progressive loss of vision resulting from optic nerve damage, often associated with increased intraocular pressure (IOP) [1, 2]. The procedures applied to decrease intraocular pressure include glaucoma medications (e.g., biomatoprost, betaxolol or levobunolol), laser therapy and trabeculectomy, which is also known as glaucoma filtering surgery [2–4]. Trabeculectomy is generally applied when medical and laser therapy have failed to sufficiently lower IOP, and in most cases, its failure is due to postoperative scarring of the filtering bleb [2–4].

Human Tenon’s fibroblasts (HTFs) are the main cells responsible for initiation and mediation of wound healing and scarring after a trabeculectomy [5]. During wound healing, activated HTFs adhere to the surgical site and start excessive proliferation and accumulation of extracellular matrix (ECM) components, leading to fibrotic scar formation [4–6]. Considerable research has focused on finding anti-fibrotic agents that would inhibit HTF proliferation and ECM production to improve the success of glaucoma filtering surgery. Agents receiving special attention have included 5-fluorouracil [7, 8], mitomycin C [7], bevacizumab [3, 9–11], and ranibizumab [4].

Because HTFs play a crucial role during wound healing after a trabeculectomy, there is a huge need to establish a simple and effective method for HTF isolation that would allow for novel ophthalmologic drug testing under in vitro conditions. From a scientific point of view, it is very important to successfully culture HTFs from trabeculectomy biopsies from patients with known treatment histories.

Before glaucoma filtering surgery, many patients take medications to decrease IOP. Moreover, anti-fibrotic agents are often administrated intraoperatively to inhibit HTF proliferation [3]. However, it is commonly observed that despite the administration of anti-fibrotic agents, postoperative scarring of the filtering bleb occurs very rapidly. This phenomenon is often associated with long-term therapy with anti-glaucoma medications [2].

Tissue biopsies taken during a trabeculectomy and further molecular analysis of HTF culture in vitro combined with the patient treatment history may provide valuable information regarding the effects of medications taken before the surgery on wound healing. As trabeculectomy biopsies are usually very small (1–2 mm in length), HTF isolation is a challenging task. It is very difficult to obtain a sufficient number of primary HTF cells from a single trabeculectomy specimen for further in vitro experiments.

**Fig. 1:**
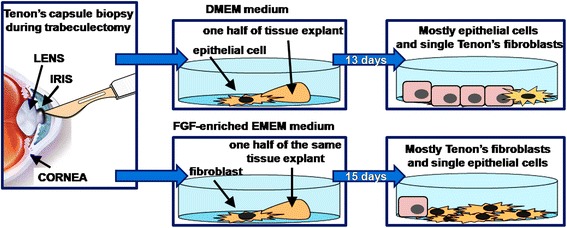
A graphic representation of the main concept of the research

The primary aim of this study was to establish a new, simple and effective protocol for HTF isolation from a single 2–3 mm × 1 mm trabeculectomy biopsy and to compare it with the standard procedure using basal DMEM, which is widely described in the available literature as a recommended medium for HTF isolation (Fig. 1). Unlike most researchers, we propose a simple “outgrowth” method without collagenase digestion, using basal EMEM supplemented with key factors for fibroblast proliferation: fibroblast growth factor, insulin and vitamin C. It is worth emphasizing that we are the first to present a fast protocol for HTF isolation from a single trabeculectomy biopsy of approx. 2–3 mm × 1 mm. The secondary goal was to compare cell morphology, size, viability, proliferation, and ability to produce type I collagen during parallel cultivation of isolated HTFs in FGF-enriched EMEM and basal DMEM.

## Methods

### Collection of trabeculectomy specimens

The Tenon’s biopsies were collected from 3 male patients (*n* = 3) aged 65–75 years who had newly diagnosed advanced open-angle glaucoma, had no previous medical therapy for the condition, and were undergoing trabeculectomy. Table 1 shows patient data. The tissue specimens were taken during the first step of the glaucoma filtering surgery. After conjunctiva separation, performed with the microscissors on the upper part of the eye bulb, a small piece of the Tenon’s capsule was harvested from the scleral area near the corneal limbus at 12 o’clock. The further steps of the surgery were typical for trabeculectomy [3, 11, 12] and did not influence specimen collection.

**Table 1:** Data about the patients from whom the specimens were collected

Patient	Sex	Age	Systemic diseases	Previous anti-glaucoma therapy
1	male	65	hypertension	none
2	male	70	none	none
3	male	75	hypertension	none

The isolated biopsy tissue, approx. 2–3 mm × 1 mm in size, was placed onto a moist tampon and transferred to a sterile 15 ml centrifuge tube containing 5 ml of 300 U/ml penicilin, 300 μg/ml streptomycin, and 0.75 μg/ml amphotericin B solution (Sigma-Aldrich Chemicals) prepared in phosphate buffered saline (PBS; Sigma-Aldrich Chemicals). Collected specimens were immediately transported to the laboratory for the isolation.

### Isolation and culture of HTFs

The tissue biopsies were washed twice with PBS and cut into 2 pieces using a sterile scalpel. These were placed in separate wells of a 12-well plate using light pressure and left to air dry for up to 1 min to attach to the well bottom.

Parallel isolations for each tissue biopsy were set up using 2 different culture media:

1. 800 μl of basal EMEM (ATCC – LGC Standards) supplemented with 5% foetal bovine serum (FBS, EU Professional grade, Pan-Biotech), 5 μg/ml recombinant human (rh) insulin, 5 ng/ml rh basic fibroblast growth factor (rh FGF b), 50 μg/ml ascorbic acid (components of Fibroblast Growth Kit, ATCC – LGC Standards), 7 mM L-glutamine (Sigma-Aldrich Chemicals), 100 U/ml penicilin, 100 μg/ml streptomycin, and 0.25 μg/ml amphotericin B
2. 800 μl of basal DMEM (Sigma-Aldrich Chemicals) supplemented with 10% FBS, 100 U/ml penicilin, 100 μg/ml streptomycin, and 0.25 μg/ml amphotericin B.

The resulting supplemented EMEM is referred to as 5% FGF-EMEM and complete DMEM is referred to as 10% DMEM throughout this article.

The explants were maintained at 37 °C in a humidified atmosphere of 5% CO_2_ and 95% air and the culture medium was renewed every 3 days. When the cells formed a monolayer, trypsinization (1–3 min at 37 °C) using 1 ml of 0.12% trypsin–EDTA solution without phenol red (Sigma-Aldrich Chemicals) was performed to detach the cells. The detached cells were then resuspended in 10 ml of fresh culture medium and transferred to 15 ml centrifuge tubes. Cells that were still attached to the culture dish surface after trypsinization were detached using a cell scraper and transferred to the same 15 ml centrifuge tubes.

The cells were centrifuged at 125 × g for 5 min, the pellet was resuspended in 5 ml of fresh culture medium, and the cells were seeded (approx. 2.4–4 × 10^3^ cells per cm^2^ of the growth area dependent on the sample) in a 25 cm^2^ T-flask and cultured at 37 °C in a humidified atmosphere of 5% CO_2_ and 95% air (passage 1)_._

When the cells reached 90% confluence, the second passage by trypsinization using 2 ml of 0.12% trypsin–EDTA solution (approx. 1–2 min at 37 °C were sufficient) was performed using two 25 cm^2^ T-flasks (seeding density of 1.2 × 10^4^ cells per cm^2^ of the growth area). The cells were also seeded in wells (1 × 10^4^ cells per well) of a 96-well plate to stain vimentin filaments.

After passage 2, fibroblasts reached confluence within 48 h. During the isolation procedure, the cell morphology was constantly monitored under an optical microscope (Olympus CKX31 or Nikon Eclipse TS100) and the obtained images were analyzed using ImageJ software.

### Recommended freezing medium and cryopreservation protocol

Two 25 cm^2^ T-flasks with confluent HTF culture from a single tissue biopsy were obtained 48 h after the second passage. The cells were then trypsinized, washed with fresh medium via centrifugation at 125 × g for 5 min and collected for cryopreservation using a freezing medium composed of 72% DMEM, 20% FBS and 8% DMSO (Sigma-Aldrich Chemicals). A Nalgene Cryo 1 °C Freezing Container was used to achieve a −1 °C per min rate of cooling. The vials (1.8 ml) in the container were placed in a −70 °C freezer for 12 h, then placed in liquid nitrogen vapour for 24 h and then in liquid nitrogen for long-term storage.

### Vimentin and F-actin filaments staining

Second passage HTF cells were seeded in the wells of a flat-bottom 96-well plate in 100 μl of complete culture medium at a concentration of 1 × 10^5^ cells/ml (1 × 10^4^ cells per well) and cultured for 48 h at 37 °C in an atmosphere of 5% CO_2_. To visualize the cytoskeletal filaments (vimentin and actin), cells were fixed according to a previously described procedure [13]. Briefly, cells were washed with PBS buffer, fixed with 3.7% formaldehyde (Avantor Performance Materials) for 10 min, permeabilized with 0.2% TritonX-100 (Sigma-Aldrich Chemicals) for 15 min and incubated with a blocking solution of 1% bovine serum albumin (BSA; Sigma-Aldrich Chemicals) for 30 min. Vimentin, a specific marker of mesenchymal cells that are highly expressed in fibroblasts, was visualized using the direct immunofluorescence technique. Cells were incubated with AlexaFluor488-conjugated mouse anti-vimentin (V9) monoclonal antibodies (Abcam) at a concentration of 1 μg/ml overnight at 4 °C. Afterwards, cells were washed with PBS buffer and simultaneously stained for 30 min at room temperature with 2 units of phallotoxin–AlexaFluor635 conjugate (Invitrogen) for F-actin filament labelling and 0.5 μg/ml DAPI (Sigma-Aldrich Chemicals) for nucleus counterstaining. To reduce nonspecific background staining, the working solution of fluorescent dyes was prepared in 1% BSA. Stained cells were observed under a fluorescence laser scanning microscope using the two-dimensional scan technique (Olympus Fluoview IV81 equipped with FV1000 laser scanner). Tenon’s fibroblasts revealed vimentin-positive (green) and actin-positive (red) fluorescence of cytoskeletal filaments, whereas epithelium-derived cells were vimentin-negative and showed only red fluorescence of actin filaments.

### Viability of the cells after thawing

After long-term storage in liquid nitrogen, the cells were rapidly thawed by placing the vials in a 37 °C water bath. Then, the viability of the cells was determined using a Countess automated cell counter (Invitrogen), which evaluates cell number and viability based on trypan blue staining.

### Parallel cultivation of HTFs in 5% FGF-EMEM and 10% DMEM

Upon thawing, the cells were washed with fresh medium via centrifugation at 125 × g for 5 min and seeded (approx. 1.2 × 10^4^ cells per cm^2^ of the growth area) in two 25 cm^2^ T-flasks. Since 10% DMEM is widely described in the available literature as a recommended medium for maintenance and propagation of isolated HTFs, the thawed cells were cultured in parallel in two different media: 5% FGF-EMEM and 10% DMEM. When the cells reached 90% confluence, they were detached by trypsinization and seeded in 96-well plates to evaluate the effect of applied culture medium on HTF morphology and size, viability, proliferation, and type I collagen production.

### Viability comparison

HTF cells were seeded in the wells of a flat-bottom 96-well plate in 100 μl of the complete culture medium at a concentration of 1.5 × 10^5^ cells/ml (1.5 × 10^4^ cells per well) and cultured for 24 h at 37 °C in 5% FGF-EMEM and 10% DMEM. Cell viability was determined by double fluorescent staining of the nuclei of dead cells with propidium iodide (red fluorescence) and the cytoplasm of viable cells with calcein-AM (green fluorescence). The dyes were the components of the Live/Dead Double Staining Kit (Sigma-Aldrich Chemicals). The staining procedure was performed according to the manufacturer’s protocol. Stained HTFs were observed under a fluorescence laser scanning microscope using the two-dimensional scan technique.

The viability of HTFs was also assessed quantitatively based on their metabolic activity using the MTT test (Sigma-Aldrich Chemicals). After 24 h incubation, 25 μl of MTT solution (5 mg/ml in PBS) was added to each well and the cells were returned to the CO_2_ incubator for 3 h. Then, formed formazan crystals were dissolved using 100 μl of 10% SDS solution (Sigma-Aldrich Chemicals) prepared in 0.01 M HCl (Avantor Performance Materials). After 12 h incubation, the absorbance was measured at 570 nm using a BioTek Synergy H4 Hybrid Microplate Reader.

### Cell morphology and size

HTF cells were seeded in wells of a flat-bottom 96-well plate in 100 μl of the complete culture medium at a very low concentration of 1.5 × 10^4^ cells/ml (1.5 × 10^3^ cells per well) and cultured for 24 h at 37 °C in 5% FGF-EMEM and 10% DMEM. Then, cells were fixed and stained using phallotoxin–AlexaFluor635 conjugate and DAPI dye as described in the *Vimentin and F-actin filaments staining* section. The morphology of the stained cells was observed under a fluorescence laser scanning microscope*.* For each sample, images were taken from 4 randomly selected fields of view and a spreading area of at least 60 individual cells was measured using ImageJ software.

### Proliferation ability

HTF cells were seeded in wells of a flat-bottom 96-well plate in 100 μl of the complete culture medium at a very low concentration of 1.5 × 10^4^ cells/ml (1.5 × 10^3^ cells per well) and cultured for 7 days at 37 °C in 5% FGF-EMEM and 10% DMEM. Every 2–3 days, the culture media were renewed. On the 1^st^, 3^rd^ and 7^th^ days of the experiment, cell number was determined based on the WST-8 proliferation test (Sigma-Aldrich Chemicals) and the calibration curve was prepared for known concentrations of cells. The test was performed according to the manufacturer’s protocol. The growth rate and doubling time of the cells were calculated using Doubling Time Computing software.

### Type I collagen production

HTF cells were seeded in wells of black, clear and flat-bottom 96-well plates in 100 μl of the complete culture medium at a low concentration of 3 × 10^4^ cells/ml (3 × 10^3^ cells per well) and cultured for 4 days at 37 °C in 5% FGF-EMEM and 10% DMEM. Then, cell number was determined based on the WST-8 test and calibration curve as described in the *Proliferation ability* section. Since WST-8 is nontoxic to the cells, the same plates were used for type I collagen (Col I) synthesis evaluation via the indirect immunofluorescence technique. The cells were fixed as described in the *Vimentin and F-actin filaments staining* section and incubated with primary goat anti-type I collagen (Col1a1/Col1a2) polyclonal antibodies (Abnova) at a concentration of 20 μg/ml (prepared in 1% BSA) overnight at 4 °C. Afterwards, the cells were washed with PBS and incubated with 2 μg/ml of the secondary AlexaFluor647-conjugated donkey anti-goat IgG polyclonal antibodies (Abcam) for 1 h at room temperature. For quantitative evaluation, the fluorescence intensity was read using a BioTek Synergy H4 Hybrid Microplate Reader with the excitation wavelength at 628 nm and emission wavelength at 670 nm (area-scan readings were recorded). The fluorescence intensity was normalized per 10^3^ cells. To visualize Col I in HTF cultures, the nuclei of the cells were additionally stained using 0.5 μg/ml DAPI. Col I production by HTFs was observed under a fluorescence laser scanning microscope using the three-dimensional scan technique.

## Results

### Isolation of HTF culture

Three parallel HTF isolations were performed using tissue specimens obtained from 3 different patients (*n* = 3) and each time the scenario was the same. Application of 5% FGF-EMEM allowed for achievement of the first monolayer in a single well of a 12-well plate after an average time of 15 days of culture (Table 2), which is very short time taking into account the single 2–3 mm × 1 mm biopsy used for the isolation.

**Table 2:** HTF isolation using 5% FGF-EMEM

Description of each isolation step	Passage number	Culture dish used	Time needed to reach monolayer	Features of obtained monolayer
Biopsy cutting and setting up of the isolation	0	Well of 12-well plate	15.3 ± 2.1 days	Mostly HTFs, possible single epithelial cells
Cell detachment using trypsin and cell scraper	1	One 25 cm^2^ T-flask	7.3 ± 0.6 days	Mostly HTFs
Cell detachment using trypsin	2	Two 25 cm^2^ T-flasks	2 days	99–100% of vimentin-positive HTFs
Trypsinization and cryopreservation of HTFs	-	-	-	-

**Fig. 2:**
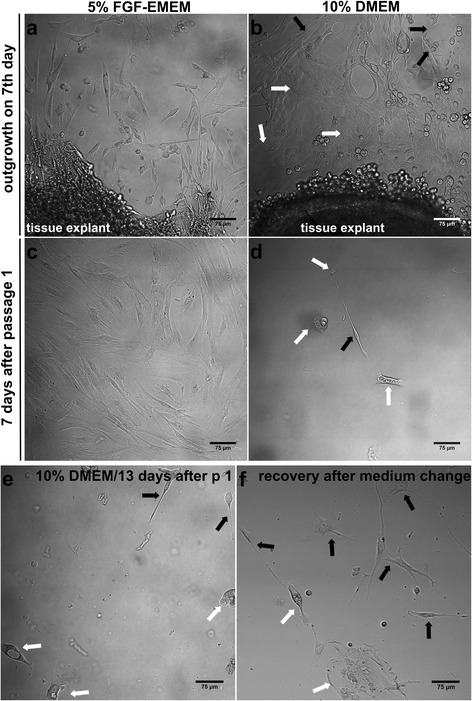
Nomarski contrast images of cells during parallel isolations from the same single trabeculectomy biopsy using 5% FGF-EMEM and 10% DMEM. **a** – Healthy outgrowth of only fibroblasts from the explant when 5% FGF-EMEM was used. **b** – Healthy outgrowth of a co-culture of predominant epithelium-derived cells (*white arrows*) and single HTFs (*black arrows*) from the explant when 10% DMEM was used. **c** – Monolayer of HTFs 7 days after passage 1 when 5% FGF-EMEM was used. **d** – Few epithelium-derived cells (*white arrows*) and single fibroblast (*black arrow*) 7 days after passage 1 when 10% DMEM was used. **e** –Few epithelium-derived cells (*white arrows*) and fibroblasts (*black arrows*) 13 days after passage 1 when 10% DMEM was used. **f** – Initial recovery of HTF culture 4 days after medium change to 5% FGF-EMEM (black arrows indicate fibroblasts, white arrows indicate epithelium-derived cells); scale bar = 75 μm, magnification 200x

More importantly, healthy outgrowth of spindle-shaped fibroblasts with individual (if any) epithelial-like cells was observed (Fig. 2a). Initial fibroblast outgrowth occurred on the approx. 4^th^ day. This concurs with De Falco et al. [14] and Gross [15], who demonstrated that initial, crucial adhesion of the cells from explants is observed between 4 and 8 days.

Similarly, a relatively short time (an average of 13 days) was required to reach first monolayer when 10% DMEM was used (Table 3). However, in the case of 10% DMEM, the monolayer of cells was predominantly composed of epithelium-derived cells and only single fibroblasts (Fig. 2b). For comparison, De Falco et al. used 10% DMEM and relatively large specimens (approx. 12 mm × 2.5 mm biopsies obtained after vitreoretinal surgery) to set up HTF culture and demonstrated that long-term incubation, approx. 20–30 days, was required to form the first monolayer [14].

**Table 3:** HTF isolation using 10% DMEM

Description of each isolation step	Passage number	Culture dish used	Time needed to reach monolayer	Features of obtained monolayer
Biopsy cutting and setting up of the isolation	0	Well of 12-well plate	13.3 ± 3.1 days	Co-culture of mostly epithelial cells and single HTFs
Cell detachment using trypsin and cell scraper	1	One 25 cm^2^ T-flask	13 days- failure	Few clusters of epithelial cells and single HTFs
Medium change from DMEM to FGF-enriched EMEM	-	One 25 cm^2^ T-flask	15.5 ± 2.1 days	Mostly HTFs and single epithelial cells
Cell detachment using trypsin	2	Two 25 cm^2^ T-flasks	2 days	99–100% of vimentin-positive HTFs
Trypsinization and cryopreservation of HTFs	-	-	-	-

**Fig. 3:**
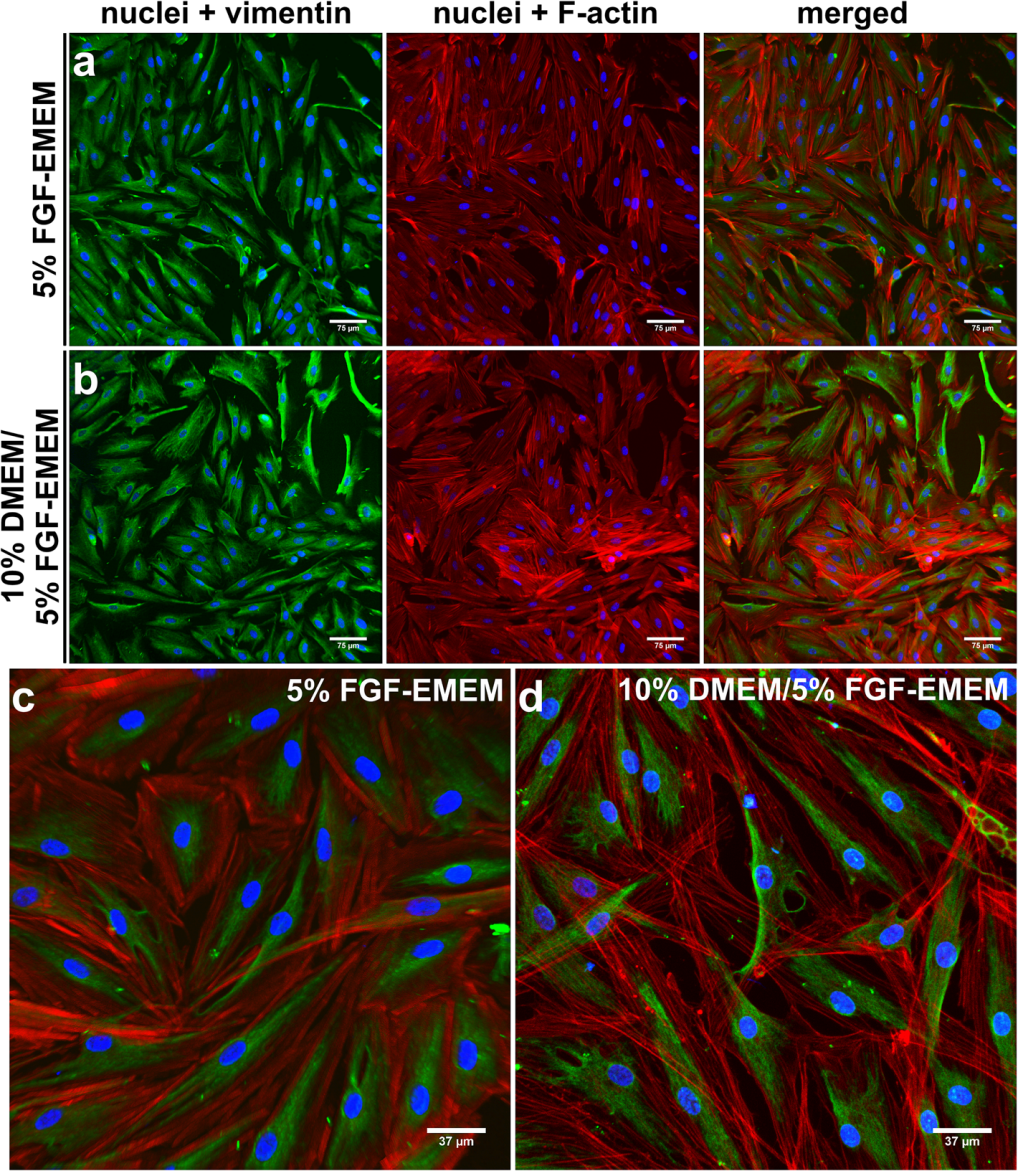
Fluorescence laser scanning microscope images of HTF cultures obtained 48 h after passage 2. **a**, **c** – HTFs isolated using 5% FGF-EMEM. **b**, **d** – HTFs isolated using 10% DMEM with medium change to 5% FGF-EMEM on the approx. 26th day. **a**, **b** have a scale bar = 75 μm, magnification 200x, while **c**, **d** have a scale bar = 37 μm, magnification 400x. Green fluorescence – vimentin filaments; red fluorescence – F-actin filaments; blue fluorescence – nuclei

When cells formed the first monolayer, passage 1 was performed regardless of the applied culture medium. After passage 1, cells reached 90% confluence (2.2 × 10^4^ cells ± 0.2 × 10^4^ cells per cm^2^) in a 25 cm^2^ T-flask within an average time of 7 days when 5% FGF-EMEM was used (Tables 2 and 4). Moreover, there were only spindle-shaped fibroblasts (Fig. 2c). On the approx. 23^rd^ day of the isolation, passage 2 was carried out using two 25 cm^2^ T-flasks and HTFs formed a monolayer within 48 h (Table 2). Immunofluorescent staining of vimentin, a specific marker of fibroblasts, revealed that near 100% of the cells after passage 2 were vimentin-positive (Fig. 3a and c).

**Table 4:** Key times during 3 parallel isolations using 5% FGF-EMEM and 10% DMEM with medium change to 5% FGF-EMEM

Type of sample	Time from biopsy to passage 1 (days)	Time from passage 1 to passage 2 (days)	Time of complete procedure, from biopsy to cryopreservation (days)
Sample 1/5% FGF-EMEM	17	7	26
Sample 2/5% FGF-EMEM	13	8	23
Sample 3/5% FGF-EMEM	16	7	25
Mean ± SD	**15.3 ± 2.1**	**7.3 ± 0.6**	**24.7 ± 1.5**
Sample 1/10% DMEM/5% FGF-EMEM	14	30	46
Sample 2/10% DMEM/5% FGF-EMEM	10	failure	failure
Sample 3/10% DMEM/5% FGF-EMEM	16	27	45
Mean ± SD	**13.3 ± 3.1**	**28.5 ± 2.1**	**45.5 ± 0.7**

Bold data are the mean values

Unlike 5% FGF-EMEM, in the case of isolation conducted using 10% DMEM, 7 days after passage 1 there was low density culture of mainly epithelial-like cells and single fibroblasts (3.9 × 10^2^ cells ± 1.3 × 10^2^ cells per cm^2^; Fig. 2d). All 3 tissue specimens (*n* = 3) were maintained in 10% DMEM for 13 days after passage 1 (Table 3). On the 26^th^ day of the isolation (13 days after passage 1) there were still a few clusters of epithelium-derived cells and only single spindle-shaped fibroblasts (Fig. 2e), so it was decided to change the medium from 10% DMEM to 5% FGF-EMEM at this point. In the case of sample number 2, medium exchange did not result in HTF recovery and the isolation failed (Table 4). In the other two samples, medium replacement resulted in rapid HTF division rates. However, the cells required at least 4–6 days to start recovery (Fig. 2f). As a consequence, fibroblasts surpassed epithelium-derived cells in growing and in an average of 15 days after medium exchange, the HTFs achieved 90% confluence (Table 3). On the approx. 43^rd^ day of the isolation, passage 2 was carried using two 25 cm^2^ T-flasks and the HTFs formed a monolayer within 48 h (Table 3). Fluorescence laser scanning microscope observation performed after passage 2 showed that over 99% of the cells were actin-positive and vimentin-positive, indicating that they were in fact fibroblast cells (Fig. 3b and d).

The efficiency of the isolation was high: the protocol enabled the isolation of 1.3 × 10^6^ vimentin-positive fibroblasts (near 100% of the cell suspension) from a single 2–3 mm × 1 mm trabeculectomy biopsy. The procedure performed for the single biopsy resulted in cryopreservation of two 1.8 ml vials and each vial contained 6.6 × 10^5^ ± 0.8 × 10^5^ cells. More importantly, the new protocol allowed the maintenance of high viability of HTFs through long-term storage (8–12 months) in liquid nitrogen (Table 5). After thawing, the cells revealed an average viability of 94% ± 2.6% (see Additional file 1).

**Table 5:** Viability of HTFs after long-term storage in liquid nitrogen

Sample no.	Time of storage (months)	Viability after thawing (%)
Sample 1	12	93
Sample 2	8	92
Sample 3	11	97

### Parallel cultivation of HTFs in 5% FGF-EMEM and 10% DMEM

**Fig. 4:**
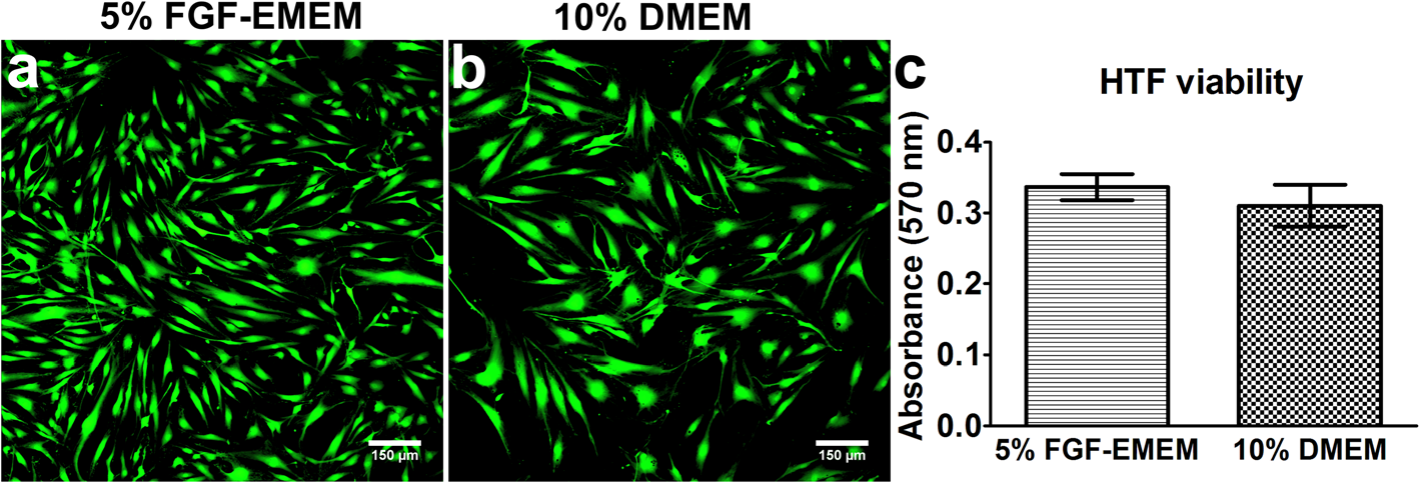
Viability of HTFs during parallel cultivation in 5% FGF-EMEM and 10% DMEM. **a**, **b** – Fluorescence laser scanning microscope images obtained upon live/dead staining of HTFs; scale bar = 150 μm, magnification 100x; green fluorescence – viable cells. **c** – HTF viability assessed based on their metabolic activity (using the MTT test). Results are expressed as means ± SD (GraphPad Prism 5, Version 5.03 Software)

Live/dead fluorescent staining followed by microscopic observation demonstrated that regardless of culture medium applied for HTF maintenance, cell viability was high (Fig. 4a and b). Microscopic images showed a high-density culture of viable HTF cells with green fluorescence. Red fluorescence indicating the nuclei of dead cells was not detected. The MTT viability test confirmed that the type of culture medium had no effect on cell viability and showed comparable metabolic activity of HTFs regardless of the medium used for cell cultivation (Fig. 4c).

**Fig. 5:**
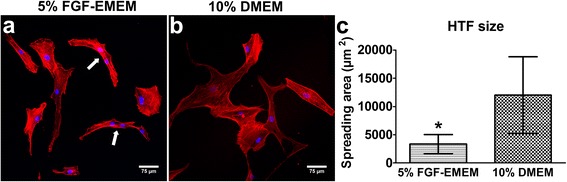
Morphology and size of HTFs during parallel cultivation in 5% FGF-EMEM and 10% DMEM. **a**, **b** – Fluorescence laser scanning microscope images obtained upon cytoskeleton staining of HTFs (*white arrows* indicate fibroblasts just after division); scale bar = 75 μm, magnification 200x; red fluorescence – F-actin filaments; blue fluorescence – nuclei. **c** – HTF size determined using ImageJ software; results are expressed as means ± SD. *Significantly lower spreading area (*p* < 0.0001) compared to 10% DMEM according to the unpaired t-test (GraphPad Prism 5, Version 5.03 Software)

Interestingly, HTF morphology, size, proliferation ability and Col I production ability were highly dependent on the applied culture medium. HTFs maintained in 5% FGF-EMEM were relatively small and had mostly spindle- or stellate-shaped morphology (Fig. 5a), whereas HTFs cultured in 10% DMEM were mostly large stellate-shaped cells (Fig. 5b). Moreover, the average size (spreading area) of the cells maintained in 5% FGF-EMEM was almost 4-fold lower (3353 μm^2^ ± 1680 μm^2^) than the spreading area of HTFs cultured in 10% DMEM (12019 μm^2^ ± 6785 μm^2^; Fig. 5c).

**Fig. 6:**
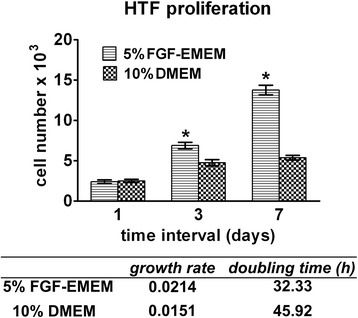
Proliferation of HTFs during parallel cultivation in 5% FGF-EMEM and 10% DMEM assessed using the WST-8 proliferation test. Results are expressed as means ± SD. *Significantly more cells (*p* = 0.0012 on the 3^rd^ day, *p* < 0.0001 on the 7^th^ day) compared to 10% DMEM according to the unpaired t-test (GraphPad Prism 5, Version 5.03 Software)

The type of culture medium used for cultivation of HTFs had also great impact on the cell proliferation ability, and thus on their growth rate and doubling time. Fibroblasts cultured in 5% FGF-EMEM revealed significantly faster proliferation and growth rates, resulting in meaningfully shorter doubling time (32.33 h) compared to the cells maintained in 10% DMEM (45.92 h; Fig. 6).

**Fig. 7:**
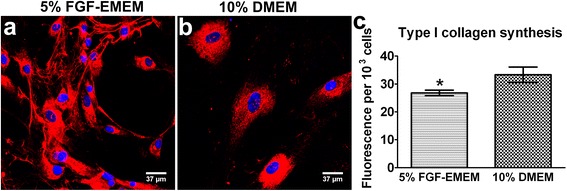
Type I collagen production by HTFs during parallel cultivation in 5% FGF-EMEM and 10% DMEM. **a**, **b** – Fluorescence laser scanning microscope images obtained upon immunofluorescent staining of Col I; scale bar = 37 μm, magnification 400x; red fluorescence – Col I, blue fluorescence – nuclei. **c** – Intensity of the Col I fluorescence (normalized per 10^3^ cells) measured using microplate reader. Results are expressed as means ± SD. *Significantly lower intensity of Col I fluorescence (*p* = 0.0007) compared to 10% DMEM according to the unpaired t-test (GraphPad Prism 5, Version 5.03 Software)

Considerable differences in Col I synthesis were also observed between the cells cultured in 5% FGF-EMEM and 10% DMEM. In the case of HTFs cultured in 5% FGF-EMEM, microscopic observation showed both intracellular formation of Col I protein and the accumulation of large and elongated collagen fibrils in the extracellular space (Fig. 7a). HTFs maintained in 10% DMEM revealed only intracellular accumulation of considerable amounts of Col I molecules (Fig. 7b). Surprisingly, despite the formation of an extensive network of Col I fibrils (a major component of ECM) by HTFs cultured in 5% FGF-EMEM, the analysis of the intensity of Col I fluorescence normalized per 10^3^ cells revealed that HTFs maintained in 10% DMEM produced slightly higher amounts of Col I than cells cultivated in 5% FGF-EMEM (Fig. 7c). Although only a slight difference in fluorescence intensity between the cells maintained in the different media was recorded, the result is considered statistically significant (*p* = 0007).

## Discussion

In this study, two different culture media were used for HTF isolation: basal EMEM supplemented with key factors for fibroblast proliferation; and the commonly recommended medium for this purpose, basal DMEM. It was demonstrated that effective HTF isolation was possible only when 5% FGF-EMEM was used. The complete procedure from the single 2–3 mm × 1 mm trabeculectomy biopsy to the cryopreservation procedure (two 1.8 ml vials) took approx. 25 days (Tables 2 and 4). Application of 10% DMEM alone resulted in isolation failure: after a 26-day culture, there were only single fibroblasts and a few clusters of epithelial-like cells (Table 3, Fig. 2e). Medium replacement to 5% FGF-EMEM at this point led to HTF recovery, but the isolation procedure (from biopsy to cryopreservation) was extended to approx. 45 days (Tables 3 and 4). It should be noted that ready-to-use specialist media enriched with FGFs and designed for optimal growth of fibroblasts would be also appropriate for HTF isolation. In our other studies, we used Quantum 333 medium (PAA Laboratories) supplemented with 5% FBS (5% Quantum 333) and we achieved comparable successful results. Similarly to 5% FGF-EMEM, the complete procedure from the single trabeculectomy biopsy to cryopreservation procedure took approx. 27 days (see Additional file 2).

According to the available literature, there are some protocols for primary Tenon’s fibroblast isolation [4, 5, 14]. Most papers present effective isolation of Tenon’s fibroblasts or trabecular meshwork cells using a complicated collagenase digestion method [1, 5] with basal culture media such as MEM [15], DMEM [1, 4, 14, 16] or RPMI-1640 [4, 17]. According to these protocols, successful HTF isolation requires long-term culture of the tissue explants: approx. 20–30 days to the first monolayer [14] unless whole eye tissue is used [1]. As trabeculectomy biopsies are very tiny (approx. 2 mm in length), most researchers describe isolation from relatively large tissue specimens obtained during other ophthalmologic surgeries, e.g. vitrectomy [14], or obtained from fibrotic scars formed after trabeculectomy [17, 18]. There are only a few protocols in the available literature describing HTF isolation from tissue biopsies obtained during glaucoma filtering surgery [15, 16].

Since FBS is a complex mixture of a large number of biologically active molecules, such as growth factors, hormones, binding and transport proteins, attachment and spreading factors, amino acids, vitamins, trace elements, fatty acids, lipids, or protease-inhibitors, the 10% supplementation of the culture medium with FBS is commonly recommended for cultivation of the vast majority of cell lines and primary cultures [19]. However, in the case of isolation of specific cell types, a serum-free culture system consisting of basal medium supplemented with growth factors, vitamins and hormones is recommended. Because serum-free media do not have any attachment/spreading factors, a pre-coating of culture dishes with components of the ECM (e.g. collagen, fibronectin) is often required. Another approach includes the use of a low concentration of FBS in culture medium (low-serum medium) instead of pre-coating of culture vessels to provide adhesive proteins (fibronectin, vitronectin, collagen), which are essential for adhesion and survival of anchorage-dependent cells [19].

In this protocol, basal EMEM was supplemented with FGF, insulin and ascorbic acid to selectively promote fibroblast proliferation. Based on the available literature, FGF promotes fibroblast proliferation and maintains the normal biological function of these cells [19, 20], whereas ascorbic acid not only stimulates fibroblast proliferation [21], but also induces enhanced type I and III collagen synthesis [21, 22]. Insulin is also known to promote fibroblast proliferation [23], as its degradation products mimic the growth-stimulatory activity of insulin-like growth factors and somatomedins [19]. FBS was added to EMEM at a low concentration of 5% only to provide essential adhesive proteins, spreading factors and trace elements. During the isolation procedure with DMEM, the FBS was the only source of growth factors, vitamins and hormones, so it was applied at a higher concentration (10%). The proliferation test, which was performed for HTFs cultured in 5% FGF-EMEM and 10% DMEM, clearly proved that addition of FGF, ascorbic acid and insulin to the basal medium significantly enhances fibroblast proliferation (Fig. 6).

Parallel cultivation of isolated HTFs in 5% FGF-EMEM and 10% DMEM revealed great differences in fibroblast behaviour. HTFs maintained in 5% FGF-EMEM were small spindle- or stellate-shaped fibroblasts with centrally placed oval nuclei, which is characteristic of active, mitotic fibroblasts [24, 25]. The cells in 5% FGF-EMEM were either fibroblasts in a late interphase just before mitosis or very small daughter cells after division, which had not reached their critical size to enter the subsequent mitosis (Fig. 5a) [26, 27]. The cells cultured in 10% DMEM were mostly large stellate-shaped cells. According to the available literature, large spindle- and stellate-shaped cells are post-mitotic fibroblasts [25] that do not have the DNA synthesis ability and thus do not enter mitosis [28]. However, post-mitotic cells possess an increased ability to produce other macromolecules, e.g. proteins [27, 28]. In this study, it was demonstrated that cells maintained in 10% DMEM stopped dividing on the 3^rd^ day of the experiment (Fig. 6) and consumed all of the available energy for protein synthesis and growth. It was also proved that HTFs cultured in 10% DMEM were almost 4-fold larger than cells cultivated in 5% FGF-EMEM (Fig. 5c) and produced relatively high amounts of Col I protein (Fig. 7c), which was not released to the extracellular space but was accumulated inside the cells (Fig. 7b). Thus, it may be inferred that large stellate-shaped cells cultured in 10% DMEM were mostly post-mitotic fibroblasts.

According to the available literature, ascorbic acid enhances type I and III collagen production by fibroblasts [21, 22]. Interestingly, in this study it was demonstrated that cells cultured in 5% FGF-EMEM produced slightly lower amounts of Col I compared to HTFs maintained in 10% DMEM (Fig. 7c). Nevertheless, unlike the cells cultivated in 10% DMEM, they revealed the ability to form large collagen fibrils, which were deposited in great amounts in the ECM (Fig. 7a and b). Thus, it may be assumed that ascorbic acid does not enhance Col I synthesis but rather induces ECM formation by promoting the excretion of collagen molecules into the extracellular space and their assembly into fibrils.

## Conclusions

The HTF isolation protocol presented here is a simple, fast method to obtain great number of cells (1.3 × 10^6^ vimentin-positive fibroblasts) from a single 2–3 mm × 1 mm trabeculectomy biopsy within approx. 25 days. Our protocol may allow for easy setup of cell banks under laboratory conditions and for novel ophthalmologic drug testing in vitro. It was also demonstrated that 5% FGF-EMEM is a better choice than 10% DMEM for fast propagation of HTFs before the preparation of the experiments. However, in the case of the studies aiming to assess the effect of a tested agent on proliferation rate or type I collagen production ability, 5% FGF-EMEM should be used only for isolation of HTFs, and then 10% DMEM should be applied for experimental setup, as 5% FGF-EMEM significantly affects cell divisions and the ECM-forming ability of fibroblasts.

## Additional files

Additional file 1: Viability of the cells upon thawing. Report generated using a Countess automated cell counter upon thawing of HTFs isolated from sample 3. The vial contained 1.8 ml of cell suspension. (TIF 5742 kb)

Additional file 2: Isolation performed with ready-to-use FGF-enriched medium. Example of another FGF-enriched medium (specialist Quantum 333) giving similar successful results. A – Nomarski contrast image of HTF monolayer 17 days after passage 1 when 5% Quantum 333 was used; scale bar = 150 μm, magnification 100x. B – Fluorescence laser scanning microscope image of HTF culture 48 h after passage 2 when 5% Quantum 333 was used; white arrows indicate single actin-positive/vimentin-negative cells; scale bar = 75 μm, magnification 200x; green fluorescence – vimentin filaments, red fluorescence – F-actin filaments, blue fluorescence – nuclei. (TIF 8732 kb)

## Abbreviations

BSA: Bovine serum albumin
Col I: Type I collagen
DMEM: Dulbecco’s modified Eagle’s medium
ECM: Extracellular matrix
EMEM: Eagle’s minimum essential medium
FBS: Foetal bovine serum
FGF: Fibroblast growth factor
HTF: Human Tenon’s fibroblast
IOP: Intraocular pressure
PBS: Phosphate buffered saline
