# Supplementary figures and images for: A simple and effective protocol for fast isolation of human Tenon’s fibroblasts from a single trabeculectomy biopsy – a comparison of cell behaviour in different culture media
Source: Cell Mol Biol Lett. 2017 Mar 9;22:5. doi: 10.1186/s11658-017-0034-4 (PMC5415760; doi:10.1186/s11658-017-0034-4)

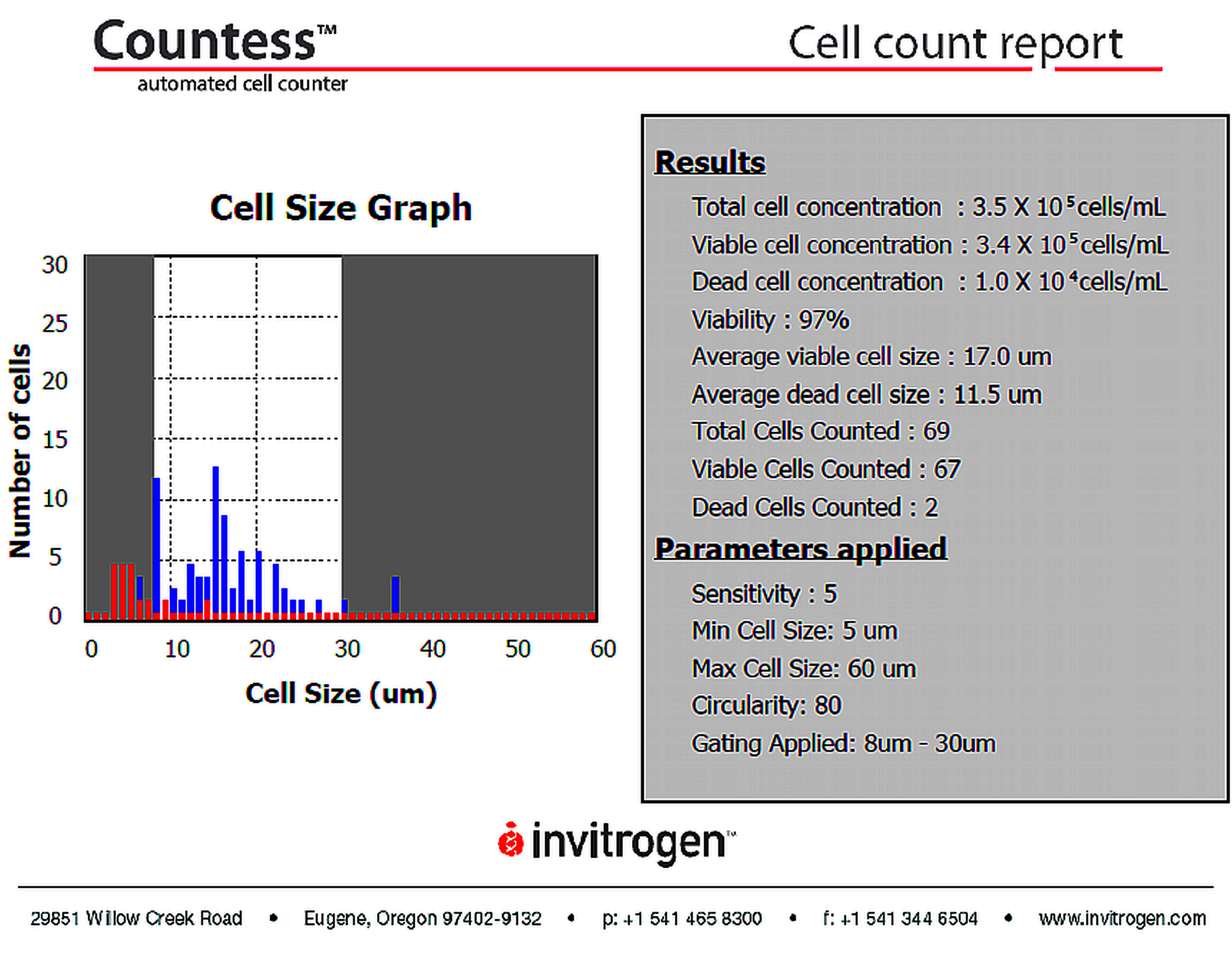

Supplement: Supplementary file 1 — Viability of the cells upon thawing. Report generated using a Countess automated cell counter upon thawing of HTFs isolated from sample 3. The vial contained 1.8 ml of cell suspension. (TIF 5742 kb) [file 11658_2017_34_MOESM1_ESM.tif]

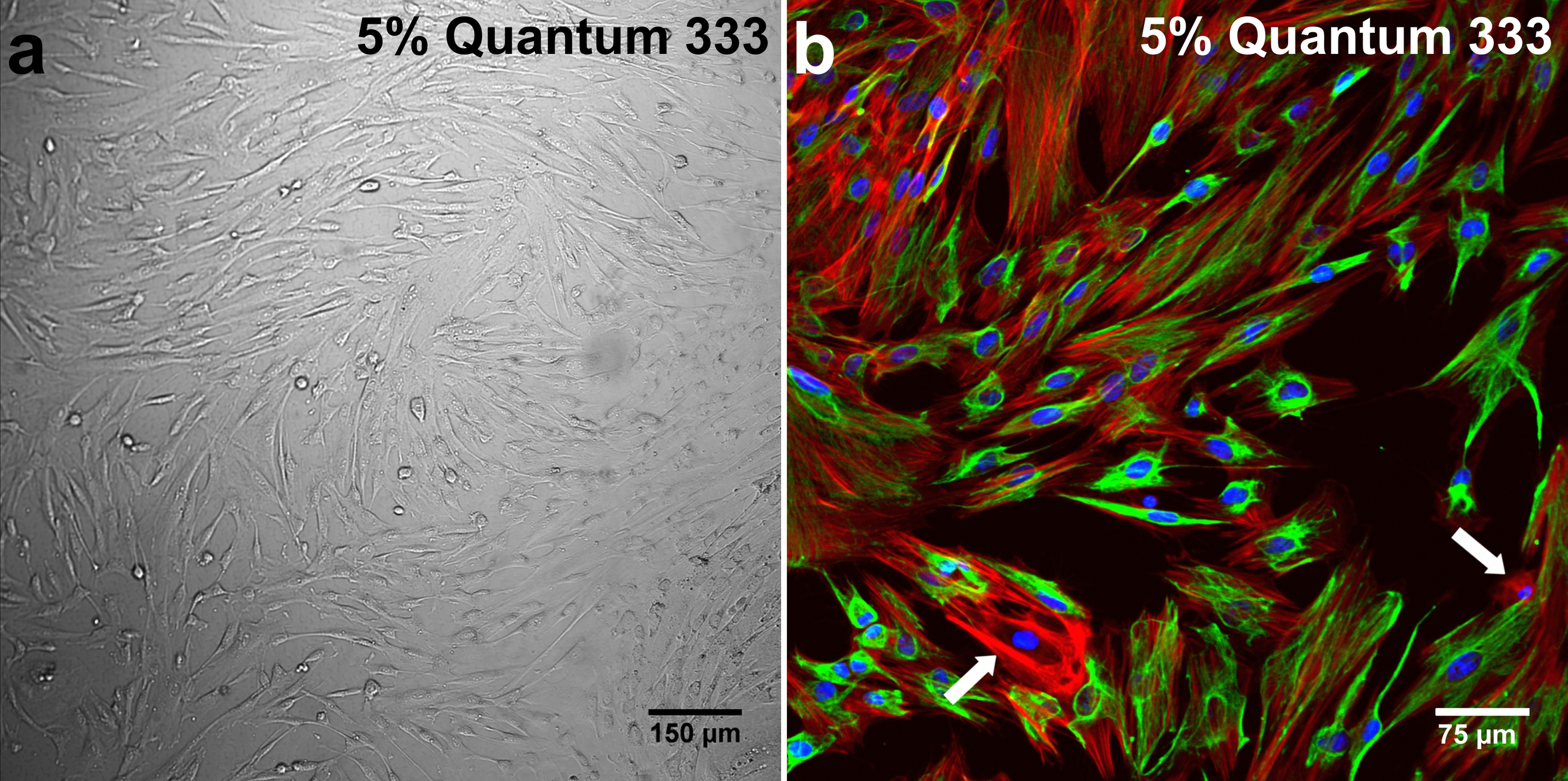

Supplement: Supplementary file 2 — Isolation performed with ready-to-use FGF-enriched medium. Example of another FGF-enriched medium (specialist Quantum 333) giving similar successful results. A – Nomarski contrast image of HTF monolayer 17 days after passage 1 when 5% Quantum 333 was used; scale bar = 150 μm, magnification 100x. B – Fluorescence laser scanning microscope image of HTF culture 48 h after passage 2 when 5% Quantum 333 was used; white arrows indicate single actin-positive/vimentin-negative cells; scale bar = 75 μm, magnification 200x; green fluorescence – vimentin filaments, red fluorescence – F-actin filaments, blue fluorescence – nuclei. (TIF 8732 kb) [file 11658_2017_34_MOESM2_ESM.tif]
